# Do entrepreneurial resources drive startup activation? Mediating effect of entrepreneurial orientation

**DOI:** 10.1016/j.heliyon.2023.e15603

**Published:** 2023-04-18

**Authors:** Zhiru Wei, Min-Jae Lee, Zhe Jia, Taewoo Roh

**Affiliations:** a School of Business Administration, Hebei Vocational University of Industry and Technology, No.626 Hongqi Street,Shijiazhuang City, Hebei Province, 050091, China; b Department of Global Business, Mokwon University, Daejeon 35349, South Korea; c Hebei University of Economics and Business, No. 47 Xuefu Road, Shijiazhuang City, Hebei Province, 050000, China; d School of International Studies, Hanyang University, 222, Wangsimni-ro, Seongdong-gu, Seoul 04763, South Korea

**Keywords:** Entrepreneurial resource, Entrepreneurial orientation, Startup activation, Global entrepreneurship monitor (GEM)

## Abstract

In order to create an entrepreneurial ecosystem at the national level, this study aims to examine the mediating role of entrepreneurial orientation between entrepreneurial resources and startup activities. Our empirical results based on samples from the Adult Population Survey (APS) and Global Entrepreneurship Monitor (GEM) data revealed that entrepreneurial resources have a positive impact on startup activation and entrepreneurial orientation plays a significant role as a mediator in the entrepreneurial resource-startup activation relationship. Our results suggest that in a business ecosystem where entrepreneurial resources persistently exist, individuals are more likely to participate in startup activation, and entrepreneurial orientation can promote startup activity not only in countries rich in entrepreneurial resources but also in emerging countries where they are scarce. Therefore, this study emphasizes the need for efforts to increase entrepreneurial orientation as well as entrepreneurial resources to create an entrepreneurial ecosystem where startups actively appear.

## 1 Introduction

In order to overcome the slowing economic growth caused by the COVID-19 pandemic, there is a growing interest in actors who encourage and strengthen new business formation and growth. In many countries, economic growth has been handled by large firms as agents under the leadership of the government [1], and this phenomenon has been noticeable in emerging markets [2]. Fostering industries centered on large firms contributed to rapid and effective economic development but caused serious problems [3]. For instance, as many small and medium-sized enterprises (SMEs) became subcontractors of large firms, their competitiveness weakened, and the polarization of the industrial structure intensified. Therefore, to reorganize the growth structure and diversify the dynamics and growth engines of the economy, there is an increasing demand to create an entrepreneurial ecosystem away from the existing national innovation system (NIS) framework [[4], [5], [6]]. According to Papaioannou et al. [7], NIS tends to value the linear input and output relationship of innovation performance, but the innovation ecosystem intervenes more strongly with market mechanisms such as interaction and network in the innovation process. Entrepreneurial ecosystems are participant-driven, with institutions regulating who acts and the outcomes of actors, in contrast to the institutional emphasis of the NIS frameworks, where governments engender and regulate action [8]. In addition to the hardware necessary for innovation, the role and value of interactions and interrelationships among innovation subjects are emphasized, and the entrepreneurial ecosystem is emerging [9,10].

The use of the term ‘ecosystem’ in the field of social science was widespread when Moore [11] introduced the concept of ecosystems to explain the increasingly dynamic and interconnected business sectors. According to Moore [11], a ‘business ecosystem’ refers to a group of businesses (or firms) and individuals that share one or more resources and coevolve together. In this context, the entrepreneurial ecosystem appears to be defined as a set of interconnected entrepreneurial actors, organizations, institutions, and processes in a particular territory [5]. Entrepreneurs are defined as innovators who develop innovation within the business ecosystem, promote economic growth, and improve the overall quality of life in society [12]. Entrepreneurial activities can make a firm more flexible by identifying business opportunities in an external environment and developing a competitive advantage in the business ecosystem based on innovation [13,14]. Therefore, entrepreneurship is considered a highly contextual process governed by country-level institutional consensus that determines its proportions and essential characteristics [15,16].

In a business ecosystem, entrepreneurship is regarded as a key driver of economic development and growth as it promotes entrepreneurial activities [17]. In this domain, a crucial role belongs to a beneficial dimension that reflects the entrepreneurial willingness to integrate technologies, resources, and new venture ideas that individuals will use in the entrepreneurial process and move toward start-ups [18,19]. For example, Hekkert et al. [20] argue that there is no such thing as an innovation system without entrepreneurship and that the role of entrepreneurs can be clarified through entrepreneurial orientation and activity. Mason and Brown [21] emphasize that entrepreneurship is an essential component of the entrepreneurial ecosystem, narrowing it to ‘high-growth start-ups’ or ‘scale-ups’ and arguing that it is a vital source of innovation, productivity growth, and employment. According to diverse scholars [5,22,23] and practitioners [24,25], entrepreneurship attracts attention as a particularly effective process for achieving sustainable and more inclusive growth in the business ecosystem.

As interest in entrepreneurial ecosystems has increased, several empirical studies have emerged from analyzing how entrepreneurial ecosystems affect entrepreneurship and subsequent value creation at the local and national levels. For example, Mack and Mayer [26] explored how a robust entrepreneurial culture and supportive public policies in Phoenix, Arizona, have consistently contributed to a strong entrepreneurial ecosystem. Spigel [23] emphasized that, while entrepreneurial ecosystems may have different structures and characteristics, their success is dependent on the ability to create cohesive socio-economic systems that support the creation and growth of new ventures. Some studies have measured entrepreneurial behavior based on quantitative data but have focused on a single national level [8,27]. Although there is a high interest in the entrepreneurial ecosystem approach, the focus on theory development [28,29] or qualitative case studies [23,30] is still dominant, and there is a lack of empirical evidence to measure entrepreneurial ecosystems with quantitative data [17]. In addition, much empirical research has almost always focused on single countries [31]. For instance, Bruton and Ahlstrom [32] focused on China's venture capital industry, and Mair and Marti [33] investigated Bangladesh. It can be more difficult for scholars to judge the impact of institutions in this environment when they only focus on single countries. In particular, due to the lack of clear empirical results for the entrepreneurial ecosystem, it may be difficult to establish appropriate support and policies to revitalize entrepreneurship from a business ecosystem perspective [5].

Meanwhile, the level of development of the business ecosystem is generally evaluated appropriately from an entrepreneurial endeavors and practices point of view [24]. Many countries fail to achieve the goal of revitalizing the entrepreneurial ecosystem and often learn from previous mistakes because they lack adequate diagnostics that inspire entrepreneurship [17]. Previous entrepreneurship research focused on using a resource-based view (RBV) to examine resources and capabilities critical to the success of new ventures [[34], [35], [36]]. Although resources are still essential, it is becoming increasingly clear that entrepreneurial orientation can affect the revitalization of the business ecosystem beyond entrepreneurial activity [37,38]. In particular, entrepreneurship is largely a regional event [28], and since there are significant differences in entrepreneurship between regions within a country [39], it is necessary to focus on the entrepreneurship orientation to analyze factors that create a successful entrepreneurship ecosystem. Therefore, by including several countries in the study, we allowed the reviewed entrepreneurial orientation to be more widely applied to developing an entrepreneurial business ecosystem.

Our research aims to unpack the structure and process for revitalizing the entrepreneurial ecosystem. Existing studies apply entrepreneur ecosystems to regional and national entrepreneurship, but the metaphors are loosely defined, theoretically undervalued, and inadequately measured [6,23]. For example, the entrepreneurial ecosystem is a system that creates successful entrepreneurship and emphasizes that access to markets, finance, and human capital are paramount to the growth of entrepreneurial firms [40]. There are many successful entrepreneurial firms, and a sound entrepreneurial ecosystem exists. However, this can be seen as the closest cause of ecosystem success, not the underlying cause, and this iterative reasoning ultimately provides little insight into research or public policy. We obtain and expand information from previous conceptual and empirical studies of the functional properties of entrepreneurial ecosystems and investigate the process of entrepreneurial ecosystem activation. To empirically verify this framework, we present empirical results obtained from databases of entrepreneurial activities in various countries. Without these studies based on secondary data from multiple countries, it is difficult to argue that institutional influences can be applied to a wide range of ecosystems and to the unusual results of samples from specific countries. This paper can contribute to developing an entrepreneurial ecosystem framework for advancing theory and policy practices.

## 2 Theoretical background and hypotheses development

The main theoretical framework of our study is the subjectivist theory of entrepreneurship [41,42]. According to this view, the prior experience and knowledge individuals receive in a particular business environment shape the direction of entrepreneurial activity and their access to resources. Individuals can prepare to launch an independent startup by raising alertness to opportunities and forming an entrepreneurial orientation based on entrepreneurial resources [43]. Entrepreneurial resources refer to the knowledge, skills, and capabilities possessed by those who contribute to entrepreneurial activity [44,45]. Entrepreneurial resources have proven to be important determinants of individual participation in startup activities, additional success in the commercialization of products or services, and business creation or upgrades [46]. More advanced entrepreneurial resources enable business opportunities to be discovered, evaluated, created, or exploited [9]. Yeganegi et al. [47] confirm that both exploratory and learning behaviors increase entrepreneurial intentions for employees of the organization in relation to emerging start-up opportunities. In other words, certain knowledge and skills acquired in the business environment create a solid foundation for individuals to switch to startup activity. According to existing literature [45,48,49], entrepreneurship utilizes personal and organizational resources and knowledge that promote learning and growth. Based on these discussions of entrepreneurial resources, we argue that entrepreneurship can open the potential of an embedded business ecosystem to a greater extent.

### 2.1 Entrepreneurial resources and startup activity

According to the RBV [50], resources determine the growth and development of a firm and are an essential component in creating opportunities for startup activity. Resources include financial, physical, and human assets and the capabilities of people in each area to formulate and implement the necessary functional goals, strategies, and policies [42,51]. In particular, entrepreneurship is based on identifying and acquiring the resources needed to start a business based on the business concepts that entrepreneurs envision [45]. Studies identifying entrepreneurship through RBV emphasize that identifying and acquiring resources is important in the early stages of new venture development [34,35]. According to Hinderer and Kuckertz [52], new ventures are created through the process of combining opportunity and resources, and in order for ventures to survive and thrive, entrepreneurs must create unique resource configurations that allow them to compete with competitors already established in the marketplace. In this vein, the individual-opportunity nexus framework suggests that entrepreneurship is concerned with the process by which founders identify, evaluate, and capitalize on opportunities to create these valuable socio-economic performances [53,54].

Several studies have shown that entrepreneurial resources (e.g., human capital or the required skills and knowledge to start a business) are important to the success and growth of startups based on these discussions [13,14]. Spyropoulos [55] argue that the knowledge of starting a business, working in the industry, and having employees internalize ways to reduce the likelihood of startup failure and enhance viability are significant. Liao, Welsch and Moutray [51] explain that the more potential entrepreneurs there are through education and training, the more innovation process activity within the enterprise is triggered, quickly identifying attractive businesses that appear in the external environment and making a firm's new ventures more flexible and challenging.

In particular, empirical evidence suggests that entrepreneurial resource retention or utilization positively affects startup performance and leads to higher entrepreneurial activity (i.e., quality and quantity of entrepreneurship) [56,57]. For instance, Wu [45] proved that entrepreneurial resources significantly affect start-up performance using data from Taiwan's high-tech firms. Huang [44] found that entrepreneurial resources further promote the pace of a startup's success through an empirical study of 374 Chinese SMEs. Consequently, entrepreneurial resources not only provide opportunities for firms but also require them for successful establishment and operation. In other words, regardless of the difference in business ecosystem scope, similarities can be drawn between the availability of entrepreneurial resources and start-up activities in that they are oriented toward intra-firm innovation, new knowledge generation, intensive growth, and significant improvement. A larger pool of resources will thus spread entrepreneurial activity, promote startup growth, and at the same time make startups more attractive to potential entrepreneurs. Taking these arguments together, we propose the following research hypothesis:

H1 Entrepreneurial resources have a positive relationship with the availability of entrepreneurial resources.

### 2.2 Entrepreneurial orientation and its mediating effect

To preserve and encourage the resources (or capabilities) that can create startups in the business ecosystem, entrepreneurs should maintain an appropriate level of entrepreneurial orientation [58]. Entrepreneurial orientation is an opportunity to develop new businesses and drive innovation [59] and is a key concept for understanding whether firms adopt entrepreneurial behavior [60]. Therefore, entrepreneurial orientation is essential to capturing entrepreneurial processes in a firm's business activities [60]. Entrepreneurial orientation guides entrepreneurs' efforts to create new ventures and develop solutions for market needs, enabling entrepreneurs to change their ecosystem structure. These challenges are particularly important in emerging markets, especially because they are often reluctant to change and primarily committed to maintaining the status quo by focusing on expanding their own markets [61,62].

Some studies have explored the determinants and performance outcomes of entrepreneurial orientation, including studies in the field of venture startups [63,64]. In particular, a stream in the venture startup literature in emerging markets focuses on the specific antecedents of entrepreneurial orientation by investigating the influence of various factors, such as family involvement and organizational and top executive characteristics, on the adoption of entrepreneurial behaviors [[65], [66], [67]]. For instance, Jaakson et al. [68] emphasize that innovation as an entrepreneurial dimension corresponds to introducing new products, services, or technology processes and that resource allocation to innovation activities is a meaningful aspect of entrepreneurship. In addition, overcoming the fear of failure is another important dimension of entrepreneurial orientation [67]. This tendency accepts risks in investment and strategic decision-making, even if the outcome is uncertain. Moreover, some scholars interpret entrepreneurial orientation as a phenomenon that occurs in group behavior [69,70]. The fundamental component here is entrepreneurial employee activity [71].

In the context of identifying multiple determinants of entrepreneurial orientation, Franco and Haase [70] emphasize that organization ownership is important to better understand entrepreneurial orientation. Ownership is the responsibility for material or immaterial objects or the existence of compulsory property rights [72]. Ownership has been regarded as a source of entrepreneurial behavior because it develops responsibility for the organization, forms awareness of a common purpose, and stimulates participation in value-creation activities [73]. Ownership is a crucial factor as a prerequisite for entrepreneurial behavior because it captures cognitive and emotional mechanisms that explain entrepreneur attachment and responsibility for business [74].

Some empirical studies emphasize the importance of ownership as a major determinant of entrepreneurial orientation. Tang et al. [75] confirmed the positive effect of entrepreneurial orientation on entrepreneurial performance based on a sample of 166 firms in northern China. More importantly, the stronger ownership is, the more positive it is for entrepreneurial performance. Liebregts and Stam [76] found that owners who impose an organizational culture based on entrepreneurship embody an entrepreneurial culture important for corporate integration and growth. In particular, ownership is related to the entrepreneurial behavior of employees because ownership can exert influence by making firms more entrepreneurial by creating conditions that increase entrepreneurship [71,77]. In other words, startups with this entrepreneurial orientation have a high ability to develop entrepreneurial activities, and as a result, they can lower the start-up failure rate by capturing survival opportunities. Based on these arguments, we propose the following:

H2 Entrepreneurial orientation mediates the positive relationship between entrepreneurial resources and startup activation relationships.

**Fig. 1:**
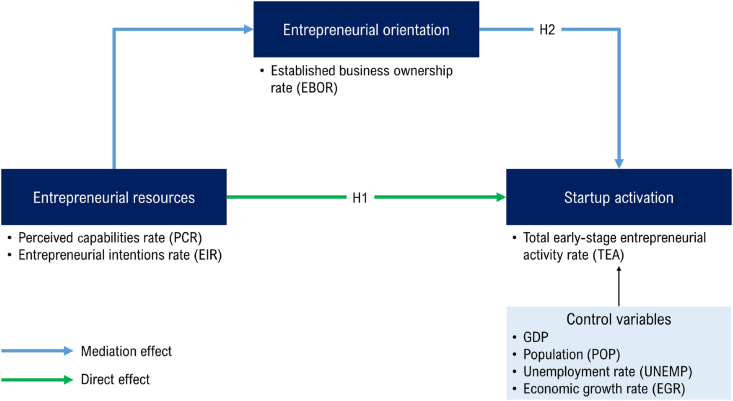
Research model.

Fig. 1 visually summarizes the hypotheses of this study discussed above.

## 3 Methodology

### 3.1 Research context and sample collection

Testing the proposed theoretical framework can be appropriately performed on multinational datasets to test the impact of entrepreneurial dimensions at the national level on the likelihood of participating in startup activities. As with previous studies evaluating national levels of entrepreneurship [57,78], the sample data for this study were extracted from the results of the ‘Adult Population Survey (APS) Global National Level Data’ conducted by the Global Entrepreneurship Monitor (GEM). Although the GEM APS dataset has its own limitations, it is considered “one of the few standardized datasets for entrepreneurial activities that enable cross-country entrepreneurship research” [79]. It has been widely used in entrepreneurship research [57,[80], [81], [82]] including investigations of entrepreneur ecosystem conditions at the national level [83,84]. Furthermore, previous studies of conducive entrepreneurial activities also typically rely on the GEM dataset [85,86]. Although many are missing, our panel data comprises a total of 2009–2020 for 115 countries, from Algeria to Zambia.

### 3.2 Variables

Dependent variables: All dependent variables were measured at the national level in 2000–2020 and extracted from the GEM's APS dataset. Following the example of prior studies of entrepreneurial activities using the GEM dataset [57], participation in startup activity was evaluated using the total early-stage entrepreneurial activity (TEA) rate in GEM, reflecting “the proportion of either nascent entrepreneurs or owners of new businesses.” The startup activity captures that a respondent is actively involved in early-stage entrepreneurial activity, including startup effort and ownership. TEA rate approximates the count of individuals currently engaged in the establishment or operation of a business, aiding in comprehending the extent of entrepreneurial activity in a geographical area or nation and pinpointing factors that can encourage entrepreneurship and contribute to economic growth development.

Mediation variables: To measure entrepreneurial orientation, the established business ownership rate (EBOR) was used in GEM [87], which reflects “the percentage of the population who is the owner-manager of an existing business.” Entrepreneurial orientation captures the percentage of the population that owns and manages an operating business that has paid its owners salaries, wages, or other payments for more than 42 months. EBOR approximates the count of businesses that have progressed beyond the initial stages of establishment, thus facilitating comprehension of the robustness of the entrepreneurial environment in a given area or nation and aiding in the identification of factors that can foster sustainable entrepreneurship and promote economic growth, thereby making it a crucial metric for policymakers, investors, and researchers invested in supporting and sustaining a thriving entrepreneurial ecosystem.

Independent and controlled variables: The primary predictor of our study is entrepreneurial resources that are closely linked to startup activity. Entrepreneurial resource availability was assessed using perceived capabilities rate (PCR) and entrepreneurial intentions rate (EIR) in GEM [88,89]. The PCR reflects the “proportion of skills and knowledge required to start a business,” and the EIR variable reflects the “proportion of potential entrepreneurs who want to start a business within three years.” PCR and EIR are crucial entrepreneurial resources as both offer a gauge of the prospective entrepreneurs’ pool, their self-assurance in their capacity to initiate and manage a business, and their aspirations to become entrepreneurs, thereby assisting in recognition of the determinants that influence entrepreneurship and directing the formulation of policies and programs that promote entrepreneurial activity. In addition, at the country level, we control for the following macroeconomic variables from the World Bank and OECD: perceived opportunities to start a business (POB), the natural log of GDP (in USD), the economic growth rate (in %), the unemployment rate (in %), and the natural log of the population [57].

### 3.3 Analytical technique

The sample used in this study is a voluntary reporting method to APS by survey respondents in each country according to year. Accordingly, it was found that the data for each year was incomplete, resulting in an unbalanced panel format. After confirming this problem and confirming that it is not a severe imbalance, we tried to verify the difference between the fixed and random effects through the Hausman test. Control and independent variables were all taken with a one-year lag. As a result of verification, the Chi2 of the Hausman test was 28.93 (*p* = 0.000), confirming that the fixed effect model was more appropriate than the random effect model. Then, pooled ordinary least squares (OLS) was attempted while controlling years to verify the robustness. All analyses in this study were validated using STATA 16©.

## 4 Results

### 4.1 Correlation results

Table 1 shows the descriptive statistics of all variables used in this study and their correlations. EIR and PCR were highly correlated with the dependent variable, TEA (0.769 and 0.687, respectively). The variance inflation factor (VIF) had a minimum value of 0.05 and a maximum value of 2.95, confirming no significant risk in the multicollinearity of our study [90].

**Table 1:** Descriptive statistics and correlations (*N* = 862).

Variables	Mean	S.D.	TEA	GDP	POP	UNEMP	EGR	POB	PCR	EIR	EBOR
TEA	11.661	7.724	1								
GDP	26.330	1.724	−0.314	1							
POP	16.782	1.625	−0.156	0.035	1						
UNEMP	7.794	5.357	−0.041	0.005	−0.105	1					
EGR	2.783	3.757	0.027	−0.031	0.113	−0.163	1				
POB	41.965	17.164	0.543	−0.187	−0.005	−0.062	0.005	1			
PCR	50.472	15.416	0.697	−0.412	−0.104	0.002	0.018	0.627	1		
EIR	20.329	15.639	0.769	−0.347	−0.138	0.023	0.012	0.519	0.667	1	
EBOR	7.656	5.027	0.522	−0.117	−0.170	−0.079	0.057	0.221	0.362	0.368	1

Noes: All correlations are significant at *p* < 0.05, TEA = total early-stage entrepreneurial activity, EBOR = established business ownership rate, PCR = perceived capabilities rate, EIR = entrepreneurial intentions rate, POB = perceived opportunities to start a business, EGR = economic growth rate, UNEMP = unemployment rate, POP = population.

### 4.2 Fixed effects model results

Table 2 shows the results of the fixed-effects model. Model 1 is the baseline that analyzes only the relationship between control variables and dependent variables. POP had a positive effect on TEA, the dependent variable, while GDP, UNEMP, and EGR had a negative impact on TEA; all of these control variables were not significant at the significance level of 0.05. In Model 2, which verifies Hypothesis 1 of this study, POB was additionally inserted as a control variable (*b*_*POB*_ = 0.027, *p* < 0.05) while holding the insignificance of the control variables used in Model 1. Both PCR and EIR as independent variables had a significant effect on TEA (*b*_*PCR*_ = 0.092, *b*_*EIR*_ = 0.234, respectively) at the 0.001 level. Hence, our Hypothesis 1 is supported. Also, in terms of explanatory power in Model 2, *R*^2^ (0.326) and adjusted-*R*^2^ (0.233) increased compared to Model 1. In Model 3, As for control variables, the negative effect of GDP on EBOR was negligible but significant (*b*_*GDP*_ = −0.000, *p* < 0.01), and all other control variables were insignificant. EBOR was changed to the dependent variable to determine whether independent variables affect our mediating variable. As a result of verification, both PCR and EIR significantly and positively affected EBOR (*b*_*PCR*_ = 0.080, *b*_*EIR*_ = 0.035, respectively). In Model 4, which verifies our hypothesis 2, we identified how the degree of influence of the existing independent variables PCR and EIR was changed while the mediator EBOR was input as a new independent variable. Control variables were all insignificant except POB (*b*_*POB*_ = 0.025, *p* < 0.05). Both PCR and EIR still significantly affected TEA even though their coefficients and significance levels were lowered, holding that EBOR is positively significant (*b*_*EBOR*_ = 0.440, *p* < 0.001), showing that partial mediation has been verified. Hence, our Hypothesis 2 is supported. The explanatory power (adjusted-*R*^*2*^) of Model 4 was 0.323, substantially improved compared to Model 1.

**Table 2:** Results of fixed effects model (*N* = 862).

Variables	Model 1	Model 2	Model 3	Model 4
DV	TEA	TEA	EBOR	TEA
GDP	−0.000 (0.000)	0.000 (0.000)	−0.000** (0.000)	0.000 (0.000)
POP	0.000 (0.000)	0.000 (0.000)	0.000 (0.000)	0.000 (0.000)
UNEMP	−0.007 (0.036)	0.042 (0.032)	−0.010 (0.025)	0.047 (0.030)
EGR	−0.007 (0.038)	−0.005 (0.032)	−0.016 (0.025)	0.002 (0.030)
POB		0.027* (0.012)	0.003 (0.010)	0.025* (0.011)
PCR		0.092*** (0.018)	0.080*** (0.014)	0.057** (0.018)
EIR		0.234*** (0.017)	0.035** (0.013)	0.218** (0.016)
EBOR				0.440*** (0.044)
Constant	11.439*** (0.396)	0.648 (0.869)	3.073*** (0.680)	0.703 (0.828)
*R*^2^	0.001	0.326	0.190	0.405
adj. *R*^2^	0.128	0.233	0.135	0.323
Log-likelihood	−2357.310	−2124.754	−1912.854	−2070.549
F	0.146***	52.195***	10.680***	64.388***
Between *R*^2^	0.000	0.702	0.155	0.773
Overall *R*^2^	0.000	0.663	0.123	0.703

Notes: Standard errors in parentheses,+*p* < 0.1, *p < 0.05, **p < 0.01, ***p < 0.001, TEA = total early-stage entrepreneurial activity, EBOR = established business ownership rate, PCR = perceived capabilities rate, EIR = entrepreneurial intentions rate, POB = perceived opportunities to start a business, EGR = economic growth rate, UNEMP = unemployment rate, POP = population.

### 4.3 Additional test

Entrepreneurship differs between developed and developing countries due to various factors [91]. Access to resources, institutional support, and market conditions are key drivers of entrepreneurship in different countries [92]. Developed countries generally have well-established legal and financial systems, providing entrepreneurs with easier access to capital, business support, and mentorship opportunities. This, in turn, allows entrepreneurs to start and grow their businesses more efficiently. On the other hand, developing countries often have weaker institutions and infrastructure, limited access to funding, and less developed markets. This makes it more difficult for entrepreneurs to start and grow their businesses. In addition, cultural and social norms may also differ, impacting the types of successful companies and the acceptable risk-taking level. Although entrepreneurship is a complex undertaking in developed and developing countries, the level of assistance and resources provided to entrepreneurs can differ greatly depending on the national economic and social progress. Therefore, examining the difference between developed and developing countries in the relationship between entrepreneurial resources, entrepreneurial orientation, and startup activation, which was examined in this study, suggests that exploring the national context to which the company belongs is meaningful.

Table 3 shows the results to verify that our hypotheses on entrepreneurial orientation can vary according to the level of national development. Regarding hypotheses 1 and 2, we sought to identify an additional question about whether the degree of entrepreneurial orientation may differ in developing and developed countries. First, based on Hypothesis 1, PCR and EIR had a positive and significant effect on TEA in developing countries in Model 1, but only EIR had a significant impact in developed countries in Model 4. In developed countries, it means that the effect of PCR on TEA is no longer exerted, so it may be desirable to form an entrepreneurial institution that fosters entrepreneurial intention rather than making prospective entrepreneurs aware of their competency. Based on H2, which verified the mediating effect of EBOR, partial mediation of PCR and EIR was confirmed in both developed and developing countries in Models 3 and 6. However, in developed countries, PCR, one of the entrepreneurial resources, significantly affected EBOR in Model 5.

**Table 3:** Additional test of fixed effects model between developing (*N* = 452) and developed countries (*N* = 410).

Variables	Model 1	Model 2	Model 3	Model 4	Model 5	Model 6
	Developing country			Developed country		
DV	TEA	EBOR	TEA	TEA	EBOR	TEA
GDP	0.803 (0.602)	−0.721 (0.456)	1.154* (0.562)	1.225** (0.374)	0.380 (0.364)	1.120** (0.361)
POP	0.226 (0.207)	−0.347* (0.156)	0.395* (0.194)	−0.069 (0.078)	0.071 (0.076)	−0.089 (0.075)
UNEMP	0.044 (0.054)	−0.037 (0.041)	0.062 (0.050)	0.006 (0.027)	−0.000 (0.026)	0.006 (0.026)
EGR	0.017 (0.062)	0.013 (0.047)	0.011 (0.057)	−0.007 (0.023)	−0.023 (0.023)	−0.001 (0.023)
POB	−0.020 (0.025)	−0.019 (0.019)	−0.011 (0.024)	0.041*** (0.009)	0.003 (0.009)	0.040*** (0.009)
PCR	0.136*** (0.030)	0.096*** (0.023)	0.089** (0.029)	0.028 (0.018)	0.074*** (0.017)	0.008 (0.017)
EIR	0.217*** (0.023)	0.037* (0.017)	0.199*** (0.021)	0.336*** (0.029)	0.080** (0.028)	0.314*** (0.028)
EBOR			0.486*** (0.063)			0.275*** (0.052)
Constant	−23.111 (15.712)	27.259* (11.896)	−36.371* (14.727)	−30.176** (9.980)	−8.697 (9.715)	−27.780** (9.638)
*R*^2^	0.319	0.094	0.411	0.491	0.136	0.528
adj. *R*^2^	0.191	−0.076	0.299	0.438	0.045	0.477
Log-likelihood	−1218.847	−1093.103	−1185.901	−748.024	−736.999	−732.727
F	25.373***	5.614***	33.053***	51.061***	8.297***	51.583***
Between *R*^2^	0.604	0.107	0.636	0.063	0.128	0.128
Overall *R*^2^	0.549	0.105	0.561	0.200	0.090	0.266

Notes: Standard errors in parentheses,+*p* < 0.1, *p < 0.05, **p < 0.01, ***p < 0.001, TEA = total early-stage entrepreneurial activity, EBOR = established business ownership rate, PCR = perceived capabilities rate, EIR = entrepreneurial intentions rate, POB = perceived opportunities to start a business, EGR = economic growth rate, UNEMP = unemployment rate, POP = population.

### 4.4 Robustness test

In addition to the above results, we verified whether the results of the panel data were consistent with OLS. Table 4 shows that the beta coefficient value and significance were almost the same, although there was a slight difference from Table 2. A partial mediating effect was still found.^2^ According to the results of the Sobel test through 20,000 bootstrapping in Table 5, the degree of the mediating effect of PCR on TEA through EBOR was 15.9%. In the same way, the degree of the mediating impact of EIR on TEA through EBOR was 11.5%. Even in the robustness test, there was no significant difference from the fixed effect model, so it is confirmed that our hypotheses 1 and 2 are still supported. Validation was attempted for an additional robustness test by proxying the dependent variable with “the number of start-up firms” provided by APS. Again, there was a slight difference in the degree of coefficient and significance, but the changes were insufficient to undermine our hypothesis in the fixed effect model and OLS regression.

**Table 4:** Robustness test of OLS regression (*N* = 862).

Variables	Model 1	Model 2	Model 3
DV	TEA	EBOR	TEA
GDP	0.000** (0.000)	0.000*** (0.000)	0.000^+^ (0.000)
POP	−0.000^+^ (0.000)	−0.000 (0.000)	−0.000 (0.000)
UNEMP	−0.080** (0.028)	−0.072** (0.028)	−0.054* (0.026)
EGR	0.030 (0.042)	0.050 (0.041)	0.012 (0.039)
POB	0.034** (0.011)	−0.021^+^ (0.011)	0.041*** (0.011)
PCR	0.159*** (0.016)	0.119*** (0.015)	0.116*** (0.015)
EIR	0.261*** (0.014)	0.041** (0.013)	0.246*** (0.013)
EBOR			0.363*** (0.033)
Constant	−2.284* (0.977)	1.566 (0.964)	−2.853** (0.914)
*R*^2^	0.688	0.200	0.728
adj. *R*^2^	0.678	0.176	0.719
Log-likelihood	−2474.032	−2462.575	−2414.799
F	70.840***	8.050***	82.723***

Notes: Standard errors in parentheses,+*p* < 0.1, *p < 0.05, **p < 0.01, ***p < 0.001, Year is included, TEA = total early-stage entrepreneurial activity, EBOR = established business ownership rate, PCR = perceived capabilities rate, EIR = entrepreneurial intentions rate, POB = perceived opportunities to start a business, EGR = economic growth rate, UNEMP = unemployment rate, POP = population.

**Table 5:** Significance testing of mediation effects.

Mediation path	PCR → EBOR → TEA		EIR → EBOR → TEA	
Method	Sobel	Bootstrap	Sobel	Bootstrap
Indirect effect	0.054	0.054	0.043	0.043
Standard error	0.007	0.007	0.005	0.005
*z*-statistic	8.109	8.079	7.815	7.797
*p*-value	0.000	0.000	0.000	0.000
Confidence interval	(0.041, 0.067)	(0.042, 0.068)	(0.032, 0.053)	(0.032, 0.054)
Baron and Kenny [93]	Partial mediation		Partial mediation	
Zhao et al. [94]	15.9% partially mediated		11.5% partially mediated	

Notes: (1) 20,000 iterations for bootstrapping, (2) confidence level of 95%, (3) TEA = total early-stage entrepreneurial activity, EBOR = established business ownership rate, PCR = perceived capabilities rate, EIR = entrepreneurial intentions rate.

## 5 Discussion and implications

This study examines the interrelationship between entrepreneurial resources and entrepreneurial orientation to realize startup activation and shows how to adopt capabilities and intentions to promote startup activities in the business ecosystem, as suggested by Mason and Brown [21]. In addition, we emphasize that entrepreneurial intentions are a catalyst for achieving startup activation. Based on the capabilities required for start-ups and entrepreneurial intentions, entrepreneurs become a means of activating start-ups through rapid entrepreneurial activities; entrepreneurial orientation catalyzes them and ultimately affects the creation of entrepreneurial ecosystems. Therefore, in order to create an entrepreneurial ecosystem where start-ups actively appear, efforts are needed to increase entrepreneur orientations as well as entrepreneur resources. In particular, since emerging markets are often reluctant to change and are devoted to maintaining the status quo mainly focusing on expanding their own markets, it is necessary to consider revitalizing startups and changing the ecosystem structure through an entrepreneurial orientation. Based on these results, we suggest the following implications.

### 5.1 Theoretical implications

Our research contributes to the study of entrepreneurship by expanding the field's understanding of specific mechanisms for entrepreneurship in start-up activities in various countries. We intended to explore how entrepreneurial resources at the national level facilitate startup activities, particularly through entrepreneurial orientation. We discuss entrepreneurial resources and entrepreneurial orientation for start-up activities in a country, deriving the following implications.

First, this study proposed an integrated framework of startup activation for entrepreneurial ecosystem construction from a subjectivist theory of entrepreneurship and recognized the relationship between entrepreneurial resources and entrepreneurial orientation. In particular, our empirical results show that individuals are more likely to participate in startup activity in a business ecosystem where quality human capital, available resources, and strong skills and capabilities continue to exist. This is a result of supporting research that argues that existing entrepreneurial resources affect startup success and revitalization [44]. Our study enriches research on how to create business using entrepreneurial resources and the impact of entrepreneurial resources on entrepreneurial success.

Second, as entrepreneurial resources are better developed at the national level, individuals with entrepreneurial orientation can foster and create innovative start-ups [46,95,96]. In particular, it shows that the desired type of entrepreneurial intention can arise from national differences, resources, and capabilities that inspire entrepreneurship [97]. Therefore, entrepreneurial orientation suggests that startup activities can be promoted in countries rich in entrepreneurial resources and in emerging countries that lack them.

Third, we have identified the critical role of an individual's ownership activities in an entrepreneurial-oriented context that creates startup opportunities. Numerous prior studies focused on research on the internal factors of organizations and individuals that promote entrepreneurial orientation. Previous studies have investigated aspects of entrepreneurial orientation that encompass individuals and organizations, such as CEO characteristics, ownership, and governance [98,99]. As such, existing evidence suggests that individual and organizational characteristics are innovative and stimulate entrepreneurship at the national level. With the current study, we add a comprehensive view of the impact of entrepreneurial orientation on the nation's start-up activities.

To sum up, our results demonstrated a positive impact on the nation's entrepreneurial resources for startup activation, and entrepreneurial orientation facilitates this.

### 5.2 Practical implications

With regard to practical implications, we demonstrate the importance of well-developed entrepreneurial resources to increase the possibility of entrepreneurial orientation in the business ecosystem and, at the same time, promote individual startup activity through entrepreneurial orientation. Emerging countries aiming for economic growth through the improvement of startup activities should invest in individual entrepreneurial capacity development, encourage challenges, and stimulate startup activities within established firms. Meanwhile, in countries where entrepreneurial resources are somewhat scarce, it is recommended that policymakers consider initiatives that can improve entrepreneurial orientation rather than expect individual entrepreneurial resources to have an immediate impact on startup activity rates.

Second, current firms should provide their employees with adequate conditions for developing entrepreneurial resources and orientation and promote environmental improvement to enhance corporate innovation further. This process can increase the proportion of innovative activities by firms and the number of innovative start-ups that can contribute to the country's economic growth. In particular, regarding managerial implications, our findings highlight the importance of adequately implementing entrepreneurship to sustain entrepreneurs who promote innovation. Thus, managers can focus on developing systematic entrepreneurial programs to maintain entrepreneurial resources and capabilities within the business ecosystem.

Third, Socio-demographic profile mapping of individuals with entrepreneurial intentions can provide insight into the process of identifying potential entrepreneurs under various government initiatives. In particular, these insights can help redirect policies and programs to build an entrepreneurial ecosystem based on targeted approaches, as emerging countries have made great efforts to revitalize startups.

These efforts can transform the business ecosystem into an entrepreneurial ecosystem and further contribute to economic development based on the promotion of startups.

### 5.3 Limitations

Our findings have several limitations. First, GEM data only captures the fact that respondents are involved in entrepreneurship and does not consider the specific characteristics that respondents possess. Admittedly, the nature of essential entrepreneurial resources can significantly affect the quality and depth of an individual's experience, which can affect additional start-up activities. Future research is to investigate not only entrepreneurial resources but also mechanisms that affect various individual entrepreneurial outcomes, such as startup intentions, startup activities, and product and technological innovations. Second, although the GEM database has provided sufficient properties for testing entrepreneur behavior, this study is primarily limited to the GEM dataset. Depending on the policy, the entire dataset of GEM will be released only three years after data collection. However, temporal changes in entrepreneurial behavior will help us better understand the new factors affecting new ventures. There is ample room for further research to develop a more robust analysis model by utilizing additional multidimensional structures for startup activation. Moreover, other characteristics of entrepreneurial orientation can be tested in future studies as mediators in the relationship between entrepreneurial resources and startup activities.

## Production notes

### Author contribution statement

Zhiru Wei; Zhe Jia: Conceived and designed the experiments; Contributed reagents, materials, analysis tools or data; Wrote the paper.

Min-Jae Lee: Conceived and designed the experiments; Analyzed and interpreted the data; Contributed reagents, materials, analysis tools or data; Wrote the paper.

Taewoo Roh: Conceived and designed the experiments; Performed the experiments; Analyzed and interpreted the data; Contributed reagents, materials, analysis tools or data; Wrote the paper.

### Data availability statement

Data will be made available on request.

### Declaration of interests

The authors declare that they have no known competing financial interests or personal relationships that could have appeared to influence the work reported in this paper.

## Footnotes

2 For verification of the mediation effect, *sgmediation2* and *medsem* command were used in STATA 16.
